# Preclinical Rat Models in Oral Implant Dentistry: A Scoping Review of Study Design and Experimental Practices

**DOI:** 10.3390/dj14060336

**Published:** 2026-06-02

**Authors:** Gian Marco Podda, Lucia Borghetti, Chiara De Siati, Paul Galvez, Umberto Romeo, Sylvain Catros

**Affiliations:** 1 Department of Oral Surgery, CHU Bordeaux, F-33076 Bordeaux, France; gianmarco.podda@uniroma1.it (G.M.P.); paul.galvez@u-bordeaux.fr (P.G.); sylvain.catros@u-bordeaux.fr (S.C.); 2 BioTis, U1026, INSERM, University of Bordeaux, F-33000 Bordeaux, France; 3 Department of Oral Sciences and Maxillofacial Surgery, Sapienza University of Rome, Via Caserta 6, 00161 Rome, Italy; desiati.1848121@studenti.uniroma1.it (C.D.S.); umberto.romeo@uniroma1.it (U.R.)

**Keywords:** rat model, preclinical research, dental implants, oral implantology, animal models in dentistry, in vivo animal models

## Abstract

**Background**: Despite the widespread clinical use of dental implants, research in implant dentistry remains active, aiming to develop new materials, designs, and surface morphologies, as well as to better understand the biological mechanisms underlying the pathophysiology of certain diseases to improve patient outcomes. In this context, preclinical animal models provide an essential opportunity to explore and validate new technologies and protocols before their application in humans. Although large vertebrate species have historically been preferred due to their biological similarity to humans, small animal models such as rats offer significant advantages. Additionally, they allow researchers to work with larger sample sizes, improving the statistical power of experimental outcomes. This scoping review aimed to analyze the current literature on intraoral rat surgical models in the field of implant dentistry. **Methods**: We included the preclinical studies using rat models focused on implant placement in the oral cavity and published in English. We excluded all studies that involved animal models other than rats or used implant placements in anatomical sites different from the target region. An electronic search was conducted in the PubMed and Scopus databases. From an initial 1032 results, 680 articles remained after duplicate removal. A first screening retained 191 articles, and after full-text review, 98 studies were ultimately included. The selection process was conducted using the software Rayyan. Data were extracted and analyzed across nine domains: Publication metadata, Focused Research Questions, Animal specificities, Study Design, Surgical Protocol Features, Medications Administered to Establish the Experimental Model, Timing of Euthanasia, Characterization methods, and Drop-Out Information. **Results**: The evaluation of the selected literature revealed a lack of standardization in study design. There is no consensus regarding the rat species used, the age at the time of implant placement, the anatomical site, or the implant morphology. Even more concerning is the presence of methodological deficiencies in the reporting of study design and outcome measures. **Conclusions**: By summarizing the available data, this review proposes the most commonly used features across preclinical trials in rats. Moreover, it offers a comprehensive overview of the current scientific landscape in this field, enabling researchers to compare different study designs and more easily access relevant information.

## 1. Introduction

Despite the widespread clinical success of dental implants [1], research in implant dentistry continues to evolve, with ongoing efforts aimed at improving biomaterials, optimizing implant design and surface properties, and understanding the biological mechanisms underlying conditions that may compromise osseointegration and long-term implant survival. Preclinical animal models are essential to validate new hypotheses and therapeutic strategies prior to their clinical translation [2].

Historically, large animal models such as pigs and dogs have been favoured due to their anatomical and physiological similarity to humans [3,4,5,6]. However, these similarities also present limitations when designing short-term experimental protocols, as larger species often require longer healing periods and more extensive infrastructure, increasing both the duration and cost of studies [7].

In contrast, rodent models, particularly rats, offer several advantages. They are cost-effective, require minimal housing space, and do not necessitate complex surgical facilities [8]. Their small size enables the inclusion of larger sample sizes, enhancing statistical power and reproducibility. In addition, the availability of a wide range of reagents and transgenic lines makes them especially valuable for molecular and mechanistic studies [9]. Moreover, rat models offer the ability to mimic systemic pathological conditions of significant clinical relevance, such as diabetes and osteoporosis, thus allowing researchers to investigate their impact on osseointegration and implant success in a controlled and reproducible manner [7]. As a result, their use in implant research has increased significantly in recent years [10].

Within rodent models, tibial and femoral sites are most commonly used for implant placement due to easier surgical access and the feasibility of placing longer and wider implants [11,12]. Nevertheless, a subset of studies has focused specifically on the oral cavity, recognizing that craniofacial bones differ from long bones in their embryological origin, healing dynamics, and mechanical environment [13,14,15]. The oral cavity provides a more clinically relevant model for dental implant research, as it introduces variables such as saliva, microbiota, and occlusal-loading factors critical to mimicking human physiological conditions.

The primary objective of this scoping review was to evaluate the current literature regarding the use of rat oral implant models in preclinical research. Specifically, we focused on studies involving implant placement within the oral cavity of rats. Secondary aims were to identify commonly adopted methodological features, to highlight deficiencies in reporting practices, and to provide a comprehensive reference framework to support future study design, data collection, and cross-protocol comparison within this increasingly utilized model.

## 2. Material and Methods

The present study was designed as a scoping review aimed at broadly mapping the methodological characteristics of intraoral rat implant models reported in the literature. The review process was conducted according to the Preferred Reporting Items for Systematic Reviews and Meta-Analyses extension for Scoping Reviews (PRISMA-ScR) guidelines [16].

No review protocol was registered for this scoping review.

### 2.1. Search Strategy

An electronic literature search was conducted using the PubMed and Scopus databases. No manual searching of “grey literature” review was performed. The search was completed on 9 March 2025. To reduce noise and improve retrieval of relevant studies, both MeSH terms and free-text keywords were used.

Search equations designed to each database were developed as follows:

- PubMed: (“dental implants”[MeSH] OR “dental implant”[TIAB] OR “oral implant”[TIAB])

AND (“rats”[MeSH] OR “rat model”[TIAB] OR “Wistar rat”[TIAB] OR “Sprague-Dawley rat”[TIAB])

AND (“surgical procedure”[TIAB] OR “implant placement”[TIAB] OR “osseointegration”[TIAB])

- Scopus: (TITLE-ABS-KEY(“dental implant” OR “oral implant”))

AND (TITLE-ABS-KEY(“rat model” OR “rats” OR “Wistar rat” OR “Sprague-Dawley rat”))

AND (TITLE-ABS-KEY(“surgical procedure” OR “implant placement” OR “osseointegration”))

In order to widen the scope of the article search, no restrictive or selective criteria were defined beforehand.

The bibliographic management software Rayyan^©^ was used for article organization and screening (https://new.rayyan.ai; accessed on 9 March 2025).

### 2.2. Study Selection

Articles retrieved from the databases were imported into Rayyan, and duplicates were removed. The study selection process was independently conducted by two reviewers (SC and GMP). Any discrepancies were resolved through discussion and consensus. Due to the broad inclusion criteria adopted in the present review, disagreements during the screening process were minimal.

The first round of screening was based on titles and abstracts. The final eligibility was determined through full-text analysis. Only studies in which dental implants were placed in the oral cavity of rats were included.

Eligibility criteria were defined according to a PICOS framework [17]:

- (P) Rat models undergoing intraoral dental implant placement;
- (I) Implant placement procedures performed in the oral cavity;
- (C) Not mandatory for inclusion;
- (O) Methodological and experimental characteristics of the implant model;
- (S) In vivo preclinical studies.

The inclusion criteria were:

- In vivo preclinical studies performed on rats;
- Studies involving dental implant placement in the oral cavity;
- Studies reporting methodological or experimental aspects of intraoral implant models;
- Articles published in English.

The exclusion criteria were:

- Studies using animal species other than rats;
- Studies involving implant placement in extraoral anatomical sites;
- In vitro or simulation studies;
- Non-English publications;
- Unavailable full-text articles.

### 2.3. Data Extraction

Data were extracted and organized into nine categories:

1. Publication metadata: Authors, year of publication, country;
2. Focused Research Questions: General and local disease modelling, surgical protocol assessment, biomaterial testing;
3. Animal Characteristics: Species, age, sex;
4. Study Design: Number of animals, experimental groups, randomization method, split-mouth design;
5. Surgical Protocol: Site of implantation, timing, material, surface, dimensions, insertion technique, healing protocol, sutures, anesthesia, analgesia, antibiotics, laser use, diet;
6. Medications associated: Bone homeostasis agents, diabetes-related drugs, fluorescent labelling, other medications;
7. Timing of Euthanasia: Number of time points, follow-up duration;
8. Characterization methods: Histology, immunohistochemistry, in vivo or ex vivo micro-Computed Tomography (µCT);
9. Drop-Out Information: Mortality rates, implant failure rates.

## 3. Results

### 3.1. Study Selection

The electronic search was performed in two databases, PubMed and Scopus, using the previously described search equations. This process yielded 1032 results; after removing duplicates, 680 articles remained. An initial screening based on titles and abstracts retained 191 articles.

Following a second assessment, based on a full-text review, 93 studies were excluded:

- Seven studies lacked full-text availability;
- Two studies were not written in English;
- Eight studies used different animal models;
- Six studies reported were in vitro experiments;
- Three studies did not include perform implant placement in bone;
- Sixty-seven studies included extra-oral bone implant placement placed implants in anatomical sites other than the oral cavity (e.g., tibia or femur).

Ultimately, 98 articles met the inclusion criteria and were selected for analysis [18,19,20,21,22,23,24,25,26,27,28,29,30,31,32,33,34,35,36,37,38,39,40,41,42,43,44,45,46,47,48,49,50,51,52,53,54,55,56,57,58,59,60,61,62,63,64,65,66,67,68,69,70,71,72,73,74,75,76,77,78,79,80,81,82,83,84,85,86,87,88,89,90,91,92,93,94,95,96,97,98,99,100,101,102,103,104,105,106,107,108,109,110,111,112,113,114,115].

**Figure 1:**
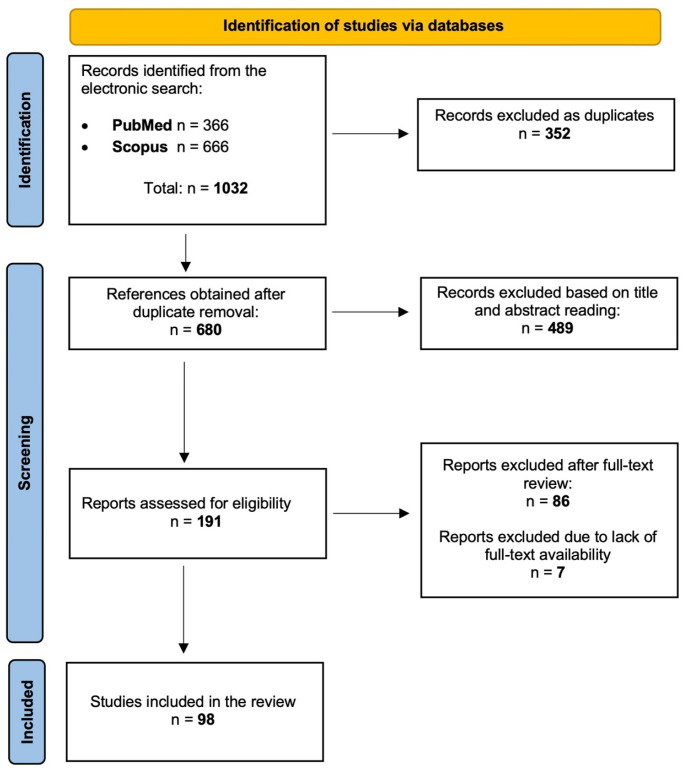
Flowchart of the study selection process for article inclusion.

The selection process is illustrated in Figure 1.

### 3.2. Publication Metadata

**Figure 2:**
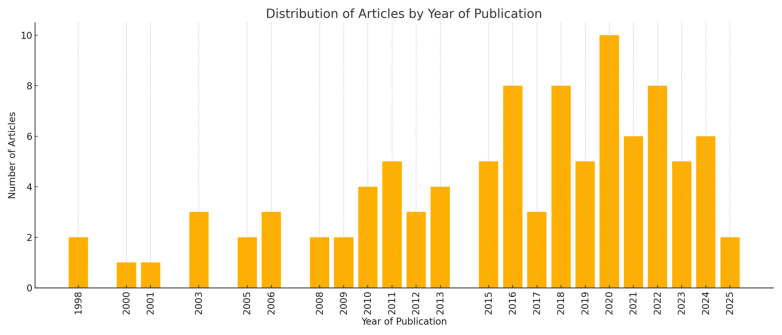
Distribution of articles by year of publication.

The studies included were conducted between 1998 and 2025 (Figure 2). Most studies (69 papers) were performed in a single country, whereas 29 were multicentric.

**Figure 3:**
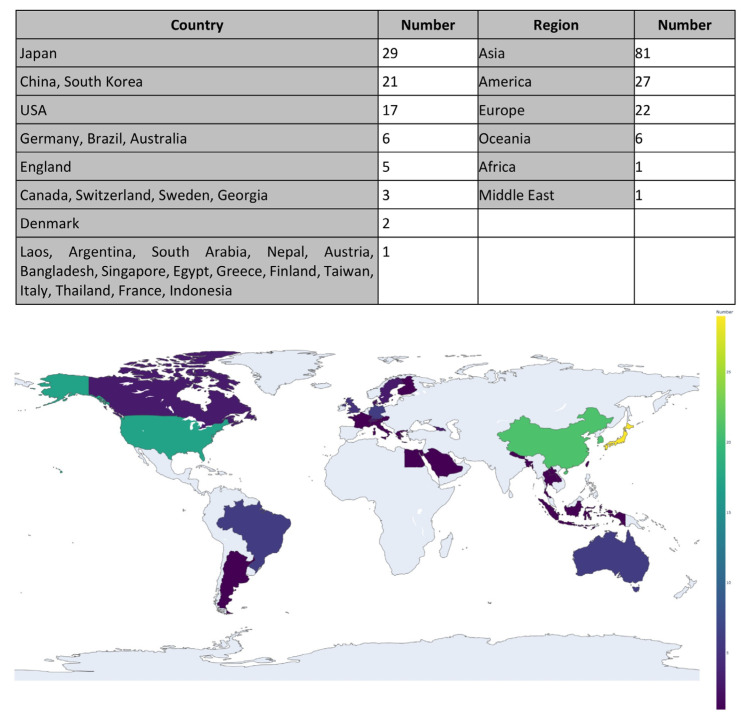
Distribution of countries involved in the studies included in the review and corresponding geographic heatmap. Each country was counted separately, including those participating in multicenter studies.

Japan accounted for 21% of the papers, followed by South Korea and China (15% each) and the United States (12%). When grouped by macro-geographical areas, Asian countries were involved in 58% of the experiments. Figure 3 summarizes the distribution of countries involved in the selected studies.

### 3.3. Focused Research Questions

A detailed scheme summarizing the focused research questions of each study is provided in Table 1. Parts of the papers focused on multiple objectives:

- Thirty-six papers established a general disease model in rats to evaluate its influence on the osseointegration process following dental implant placement. Specifically, in 15 papers, implants were placed in an osteoporotic rat model [24,36,37,40,41,50,51,55,68,70,72,82,94,95,96], while in 3 studies, the osteoporosis model was combined with the simultaneous administration of antiresorptive medications (bisphosphonates) [53,58,115]. Bisphosphonates were also administered in six additional experimental trials alone [52,66,81,91,98,99] or in combination with stress behaviour factors [29]. In ten papers the authors evaluated the osteointegration process in a diabetic rat model [18,32,33,49,54,73,84,103,104,109]. Other objectives were to evaluate the effects of radiotherapy [92] or stress on osseointegration [97].
- Twenty-four studies developed a local disease model in which implants were placed. In 13 papers, occlusal loading was applied to evaluate its impact on bone structure [19,24,28,31,39,42,45,49,56,65,67,71,100]. In seven papers, a peri-implant disease model was created using bacteria-loaded ligatures or direct contamination of the implant surface [23,38,62,63,83,110,114]. Additional studies examined the impact of bone heating [35,88] or alveolar nerve injury [64,74] on healing.
- Twenty-six experimental protocols aimed to evaluate how various surgical parameters affect osseointegration. The authors evaluated how the implant surface and the implant morphology as well as the implant material could affect the healing process in rats. A part of them, specifically nineteen papers, compared different implant surfaces [22,23,26,27,37,43,54,55,63,66,78,79,87,90,93,98,100,109,114] while four evaluated different implant materials [52,85,86,89]. Only two studies focused on implant morphology [35,101], and 1 tested different healing times [50].
- Eleven studies investigated the use of biomaterials in rat models. Collagen sponges represented the most frequently employed material, reported in three studies either as a standalone scaffold [38,76] or in conjunction with platelet-derived growth factor (PDGF) [46]. Bone morphogenetic proteins (BMPs) [80,102] and autogenous bone grafts [30,75,77] were each used in two studies. Other materials, reported in single studies, included α-tricalcium phosphate (α-TCP) [59], a hybrid construct [110], and a basic space-maintaining device [69].

Some studies addressed more than one research aim and were therefore classified in multiple categories within Table 1.

In addition, seventeen studies [20,21,25,34,44,47,48,57,60,61,105,106,107,108,111,112,113] did not fit into any of the predefined categories and were therefore grouped under the category ‘Other aims’.

### 3.4. Animal Characteristics

The most commonly used rat species were Sprague-Dawley (63%) [20,21,23,25,26,27,28,30,31,33,34,35,36,37,38,39,41,42,43,44,46,47,50,51,52,53,54,55,57,58,61,62,63,64,66,70,72,75,76,81,82,84,85,86,87,90,91,92,93,94,95,96,98,99,102,104,109,110,111,114,115] and Wistar (33%) [18,22,24,29,32,40,45,48,56,59,65,67,68,69,71,73,74,77,78,79,80,88,89,97,100,101,103,105,106,107,108,112,113], with other strains such as Lewis marginally represented (2%) [19,83]. One study did not specify the species used [49].

**Figure 4:**
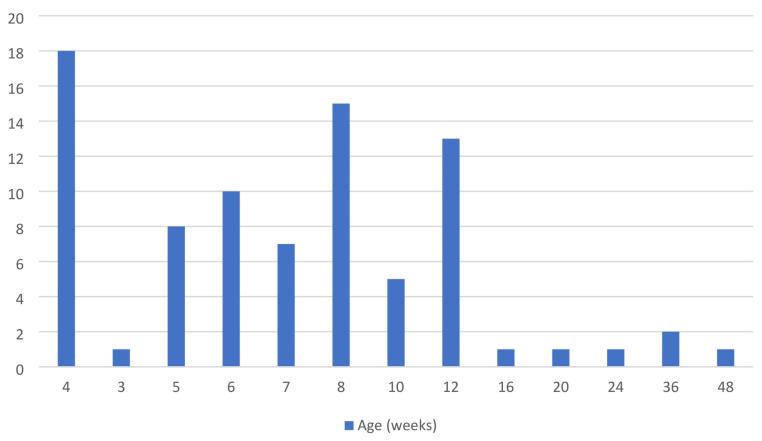
Distribution of rat age at the beginning of the experiment.

The age at the beginning of the experimental protocol typically ranged from 6 to 12 weeks (Figure 4), with a mean of 8.4 ± 6.6 weeks.

Males were used more frequently than females (62% vs. 25%). Only one study included both sexes, and 11 did not report the gender of the animals.

### 3.5. Study Design

On average, 2.5 groups were included per study, typically comprising one control and one or more test groups. Eighty percent of studies included a control group.

A randomized design was reported in 42% of studies, while 12% employed a split-mouth approach.

The average number of enrolled animals per study was 33 ± 17.

### 3.6. Surgical Protocol Features

The first maxillary molar region was the most frequently used site for implant placement, appearing in 70 studies [18,20,21,22,23,24,28,29,30,31,32,33,35,36,38,39,41,42,44,45,46,47,49,50,51,52,53,55,56,57,59,60,61,62,63,66,67,68,70,71,72,75,76,78,80,81,82,84,85,86,88,89,91,92,94,95,96,98,99,100,101,102,103,104,106,107,108,111,112,113]. The mandibular first molar was used in 12 cases [26,27,37,40,43,54,64,74,87,90,93,109]. Fewer studies explored non-molar sites in the maxilla (e.g., anterior region, hard palate; 8 studies) [19,25,34,58,83,110,114,115] or mandible (e.g., ramus, angle, or lateral body; 5 studies) [48,69,77,97,105], with even fewer targeting the mandibular incisor (2 studies) [73,79] or second maxillary molar (1 study) [65].

A bilateral placement approach was used in 43 studies [18,21,23,27,30,32,33,35,36,44,45,46,50,52,55,56,57,66,67,68,72,74,76,78,80,81,82,83,84,85,86,88,91,92,94,95,99,102,107,108,110,111,112,113].

Regarding timing, thirty-four studies adopted an immediate placement protocol post-extraction [20,23,30,33,35,41,44,47,52,55,63,64,65,66,71,73,74,75,79,80,82,84,85,86,89,95,96,98,99,100,101,103,104,106], while the remaining studies followed a delayed approach, with an average healing period of 32.0 ± 14.9 days. Specifically, thirty-three studies employed a 28-day healing interval [21,22,26,27,28,31,32,36,37,38,43,45,46,49,51,54,56,57,59,60,61,62,67,70,76,87,88,90,92,93,94,109,113], which emerged as the most common delayed protocol. The longest healing time reported was 112 days. In thirteen studies, no tooth extraction was necessary, as implants were placed directly in anatomical sites, such as edentulous regions of the maxilla or mandible, where extraction was not a prerequisite (e.g., mandibular angle, palate mesially to the first molar).

Titanium and its alloys were the materials of choice in nearly all studies. Two studies did not report the material used. Zirconia was used in four studies that specifically aimed at evaluating the performance of different implant materials [52,85,86,89].

Regarding implant surfaces, 41 studies did not specify the type, and 19 compared different surface types [22,23,26,27,37,43,54,55,63,66,78,79,87,90,93,98,100,109,114]. Among the remaining studies, 17 used rough surfaces [20,38,44,52,56,61,62,69,76,81,82,85,86,88,97,102,111], 15 used machined [21,24,45,57,75,77,80,89,91,99,101,106,107,113], 4 used coated [40,50,71,103], and 2 used anodized surfaces [33,84].

**Figure 5:**
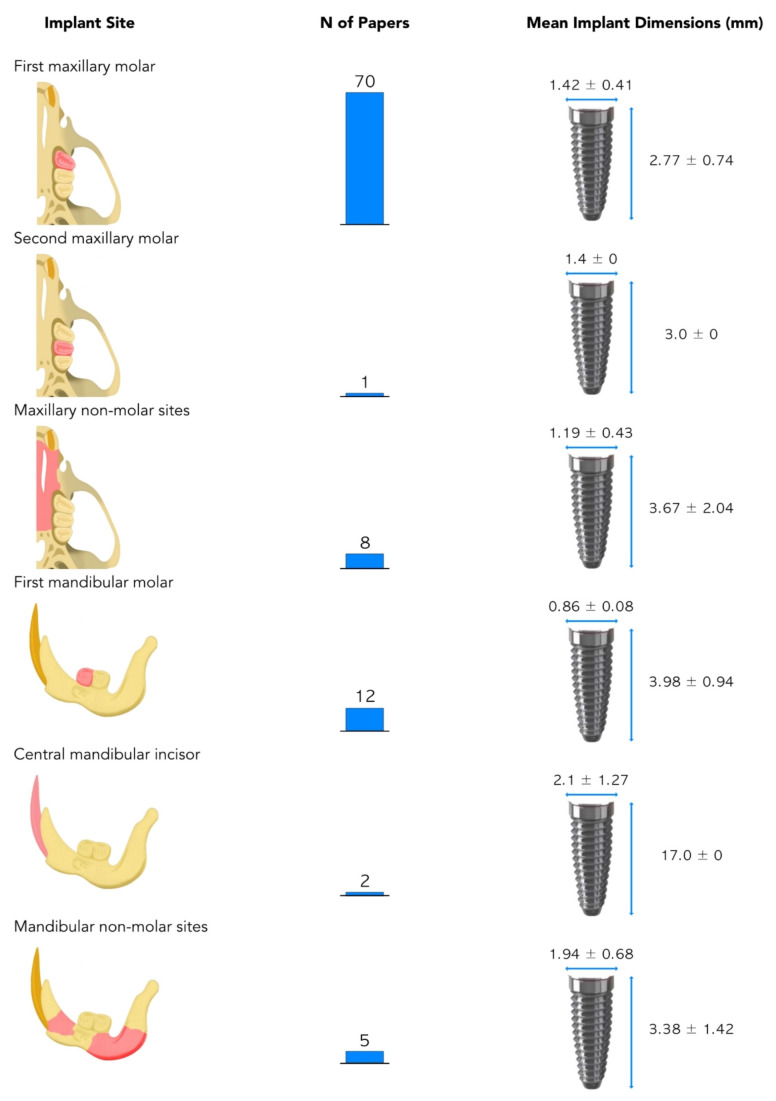
Summary of implant site selection and implant dimensions (diameter and length) across included studies.

Implant dimensions varied by site, primarily reflecting differences in available bone width and density. Figure 5 summarizes these values and shows the implant site distribution.

In the maxillary first molar site, the average diameter and length were 1.42 ± 0.42 mm and 2.77 ± 0.74 mm, respectively, showing low variability and standardization. The second maxillary molar site, reported in one study, had similar dimensions (1.4 mm diameter, 3.0 mm length). Implants in the mandibular first molar site had the smallest diameters (0.86 ± 0.08 mm), which is attributable to the limited buccal–lingual space in the molar region of the rat mandible. The narrow standard deviation further reflects the low variability in implant diameter. The average length reported for this site was 3.98 ± 0.94 mm. The mandibular incisor site had a uniquely long implant length of 17.00 ± 0 mm and a diameter of 2.10 ± 1.27 mm, reflecting site-specific anatomy. Mandibular non-molar sites supported larger and longer implants (1.94 ± 0.68 mm diameter, 3.38 ± 1.42 mm length), likely due to thicker cortical bone. Maxillary non-molar sites showed high dimensional variability (1.19 ± 0.43 mm diameter, 3.67 ± 2.04 mm length).

Most of the studies (68 papers) used screwed implants [19,20,21,24,26,27,28,29,31,32,34,35,36,37,38,39,41,42,43,44,45,47,49,51,52,53,54,55,56,57,58,60,62,63,65,66,67,68,69,70,71,72,73,74,75,77,79,85,86,87,88,89,90,92,93,94,95,96,97,98,101,103,104,105,106,109,110,115] while nineteen papers used press-fit implants that were inserted passively into undersized osteotomies [18,40,46,48,59,61,76,78,80,81,82,91,99,100,102,107,111,112,113]. The insertion method was not detailed in 11 of the included studies.

Postoperative healing was transmucosal (51 studies) [20,21,22,23,24,26,27,28,29,30,31,33,35,39,41,42,43,47,49,51,52,53,54,56,58,60,63,65,67,68,70,71,72,79,84,85,86,87,89,90,92,93,94,98,100,101,103,106,109,110,114] or submerged (33 studies) [18,32,38,44,45,46,48,55,57,59,61,62,66,69,74,75,76,77,78,80,81,82,83,88,91,96,97,99,102,105,107,111,113]. It was not specified in 14 studies.

Sutures were not used in 62 articles, and this was predominantly aligned with transmucosal healing (94% of these studies did not apply sutures). Conversely, 22 studies employed traditional suturing techniques [18,42,44,48,55,56,57,58,59,62,66,69,75,78,80,81,82,88,96,105,107,113], and 6 opted for tissue adhesives, such as Periacryl©, to close the surgical site [38,46,61,76,102,111]. Eight papers did not report whether sutures or any closure method were used.

The surgeries were done under general anesthesia, using injectable anesthetics (*n =* 67) [18,21,25,26,27,30,31,33,34,35,36,37,38,40,42,43,47,48,49,50,51,52,53,54,57,58,59,61,62,63,64,65,68,69,70,71,72,74,75,76,77,78,83,84,85,86,87,88,90,91,92,93,94,97,99,100,101,102,103,105,106,107,108,109,111,112,113] or inhalated isoflurane (*n =* 8) [32,39,44,55,66,82,98]; anesthesia type was not specified in 23 protocols. The most commonly used injectable anesthesia was a solution of Ketamine and Xylazine (31.3%). Zolazepam-based protocols, either in combination with Tiletamine and Xylazine or with Xylazine alone, were also commonly used (22.4%).

Local anesthesia was administered in some reports (*n =* 8), using lidocaine [21,31,39,57,60,83,91,99].

Postoperative analgesia was underreported (not mentioned in 70 studies). Among the minority of studies that did use analgesics (28 papers) [30,31,32,35,36,39,44,48,50,51,52,53,55,60,62,66,70,72,76,81,82,85,86,94,97,98,101,115], Buprenorphine administration alone (8 papers) or in combination with Carprofen (5 papers) or Ketoprofen (3 papers) was common.

Antibiotic prophylaxis was not standardized. Sixty-nine studies did not report antibiotic use. When used, beta-lactams were the most common (*n =* 9), including penicillin, ampicillin, or amoxicillin [46,61,75,76,81,97,100,102,115]. Eight trials employed aminoglycosides, such as gentamicin and amikacin [21,27,51,53,57,70,94,109]. Other medications employed included fluoroquinolones [44,55,66], tetracyclines [110] or combination therapies [33,82,84], such as ampicillin paired with gentamicin or enrofloxacin.

Perhaps most concerning is the proportion of studies that simply stated the antibiotic use without specifying the drug (*n =* 5) [36,43,54,87,93].

The use of a soft diet as a postoperative management strategy was observed in only 13 studies [19,31,32,39,57,59,77,80,81,82,101,106,113], while the vast majority (85 papers) did not report any dietary modification following implant placement. Only one paper reported the administration of low-level laser therapy after implant placement to promote healing [63].

### 3.7. Medications Administered to Establish the Experimental Model

Eighteen papers used pharmacological interventions to modify bone metabolism. Bisphosphonates were the most common class (11 studies) [29,52,53,58,66,85,86,91,98,99,115], with alendronate administered in 9. Other drugs included Parathyroid hormone (PTH) (3 studies) [70,77,94], Raloxifene (*n =* 2) [36,51], and Abaloparatide (*n =* 2) [81,82].

Five studies employed fluorochrome-based dynamic markers such as Calcein or Alizarin red S [46,55,92,102,104].

Fluorochrome labeling was rarely used, appearing in only five studies (e.g., Calcein, Alizarin Red S).

Three studies addressed systemic diabetic alterations using Insulin, Metformin, or Advanced glycation end products (AGEs) [32,41,49].

Eight studies administered other agents, including simvastatin and clodronate liposomes [21,25,30,38,57,64,72,95]. Although clodronate is a bisphosphonate, when encapsulated in liposomes with phospholipids (clodronate liposomes), it selectively depletes macrophages.

The detailed results for this domain are detailed in Table 2.

### 3.8. Timing of Euthanasia

Euthanasia timing was recorded in days, calculated from the day of implant placement to the day of euthanasia.

A single endpoint was used in most of the cases (34 papers), but we also observed several studies including two different endpoints (26 papers).

More sophisticated designs using three or four time points were less common (11 papers each). Eleven papers designed trials with more than five different time points for euthanasia, while five studies did not report the timing of euthanasia.

The data revealed considerable heterogeneity in euthanasia timing across the 98 studies analysed: the mean euthanasia time was 50.2 days (SD = 52.4 days).

The most common endpoint was 28 days (32 papers), but 56 days was also frequently used (16 studies).

### 3.9. Characterization Methods

Histological analysis of the samples was performed in most of the studies included (80 out of 98). The majority of these studies (*n =* 44) removed the implant before histological processing [21,22,26,27,28,29,31,33,34,35,37,41,42,43,49,50,54,56,57,58,59,61,63,67,70,71,72,73,78,79,83,84,87,88,90,91,93,99,101,106,108,109,113,114]. A smaller subset (*n =* 29) conducted histological analysis with the implant in place [18,20,32,38,44,46,48,51,52,53,55,62,65,66,69,74,75,76,77,81,82,94,95,96,97,100,102,110,115]. In some cases, (*n =* 6 papers) both types of analysis were reported [24,45,68,92,103,104].

Removing the implant enabled immunohistochemical evaluation of the samples. Specifically, among the 41 trials in which immunohistochemistry was performed [18,21,22,24,26,27,29,31,35,36,37,39,42,43,47,49,50,54,56,57,59,61,64,67,71,72,73,78,79,84,87,89,90,93,99,101,107,109,112,113,114], the implant was removed in 33 cases.

The second method used to analyse the results was micro-computed tomography (μCT), a high-resolution imaging modality essential for evaluating qualitative and quantitative peri-implant bone parameters. It was performed in 45 studies [19,23,25,26,27,29,32,33,34,35,37,38,41,43,46,50,51,53,54,56,57,63,65,66,67,72,75,76,81,82,83,84,86,87,90,91,92,93,94,98,100,109,110,111,114], mostly as ex vivo µCT (*n =* 41).

### 3.10. Drop-Out Information

Only 22 studies reported the percentage of animal loss during the experimental period [18,21,29,32,36,48,55,58,66,67,69,74,77,80,85,86,88,91,92,96,99,110]. The mean mortality rate across these studies was 3.35%, with a standard deviation of 4.61%.

A slightly larger number, 30 studies, reported the percentage of implant failures [18,21,32,33,36,45,48,52,55,56,57,58,62,66,67,69,74,77,80,85,86,88,91,92,93,96,97,98,99,115]. The average failure rate was 10.85% (SD = 13.47%), indicating high variability among studies.

## 4. Discussion

This scoping review provides a comprehensive synthesis of 98 preclinical studies using rat models in the field of oral implantology. Among rodent species, this review focuses on rats rather than mice due to the significant differences in tooth dimensions between the two. In fact, mice exhibit linear tooth dimensions that are approximately half those of rats [116], making surgical procedures even more technically challenging. Given that implant placement in the rat oral cavity is already demanding, rats represent a more suitable model for such preclinical studies.

The review identified significant variability across nearly every domain of study design, including animal characteristics, surgical protocols, and outcome assessment methods. Despite this heterogeneity, certain patterns emerged, enabling the identification of commonly used features.

Analysis of the geographical distribution of the selected studies reveals a predominance of contributions from Asian research institutions in the domain of preclinical dental implant research utilizing rat models. This pattern may be attributed to regional scientific priorities, differential funding strategies, and the presence of dedicated infrastructure for in vivo animal studies [117].

Sprague-Dawley and Wistar rats dominated the included studies, consistent with their established role in biomedical research due to favourable temperament, size, and availability of immunological and genetic data [118,119]. The mean reported age of the rats at the beginning of the experiment (8.4 ± 6.6 weeks) corresponded to young adult rats, which are ideal for osseointegration studies due to active metabolism and the absence of senescence-related changes [120]. Notably, a preference for males was observed, because of possible estrogen-related hormonal effects on bone homeostasis seen in females [91]. However, this practice underscores an ongoing controversy in preclinical research design, as the exclusion of female subjects may compromise the reproducibility and translational relevance of the results. Inclusion of both sexes is increasingly recognized as essential to ensure that preclinical findings are robust and broadly applicable [121].

Most studies involved approximately 30–35 animals and included two to three experimental groups, with at least one control group. The mean sample size observed is consistent with those commonly reported in in vivo dental implant studies. The observed high standard deviation in both sample size and group distribution across studies is most likely a consequence of the heterogeneity in study objectives and experimental designs. While randomization was reported in 42% of studies, only a minority used split-mouth designs, which could help reduce inter-animal variability.

The first maxillary molar site emerged as a standardized anatomical location, used in over 70% of studies. Its anatomical consistency, accessibility, and compatibility with standardized implant dimensions (mean diameter: 1.42 mm; length: 2.77 mm) make it a reliable choice for preclinical implant studies [44]. Nevertheless, certain anatomical concerns persist, including the risk of root fracture during extraction and the potential for maxillary sinus perforation during drilling and implant placement [122].

An additional limitation of intraoral rat models is represented by the reduced implant dimensions required by the anatomical constraints of the oral cavity. These dimensions differ substantially from those commonly used in clinical implant dentistry and may influence primary stability and peri-implant healing dynamics. However, similar dimensional adaptations are also necessary in extraoral rodent implantation models because of the anatomical limitations inherent to small animal models [123]. Moreover, intraoral implant placement in rats remains technically demanding and may be associated with limited surgical access, risk of root fracture during tooth extraction, possible sinus perforation in maxillary sites, and variability in occlusal loading conditions. Nevertheless, despite these limitations, intraoral models provide the opportunity to investigate implant healing within a biologically relevant oral environment characterized by saliva exposure, oral microbiota, alveolar bone remodeling, and masticatory-related factors that cannot be adequately reproduced in extraoral implantation sites.

Fewer than half the studies (*n =* 43) implemented a bilateral approach. This method aligns well with the 3R principles in animal research [124], facilitating statistical power while reducing the number of animals used.

Although both immediate and delayed implant placements were common, almost one-third of studies used a 28-day healing period post-extraction [125], which aligns with critical bone remodelling phases in rodents. Immediate placement may increase complication rates; for example, Khina et al. [86] reported a high implant failure rate (~40%) using this protocol. Extraction surgery in rats is technically challenging and may result in root fractures, necessitating more invasive procedures and jeopardizing primary implant stability [126].

Titanium, in pure or alloyed form, was by far the most commonly used implant material, reflecting its established role as the gold standard in both clinical and preclinical implantology. Although zirconia presented an alternative [127], only four studies reported its use, likely due to the difficulty and cost of fabricating small zirconia implants.

Despite the known relevance of surface properties on osseointegration, surface characteristics were underreported in 41% of studies. However, nineteen studies specifically evaluated this variable.

Screwed insertion protocols were predominant, reflecting a preference for mechanical stability.

Transmucosal healing protocols were employed in over half of the studies. Their prevalence may be due to manufacturing simplicity, as the transmucosal neck adds ~1–1.5 mm to implant length, and procedural ease. This method correlated strongly with the omission of sutures (94%), reflecting simplified surgical protocols. Submerged healing, though less common, may have been avoided due to surgical complexity. On the other hand, exposure of implants to the oral environment presents a significant risk to bone healing, potentially compromising osseointegration through microbial contamination, salivary enzymes, and mechanical disturbances [128].

Anesthetic protocols varied widely. Injectable combinations, particularly ketamine and xylazine, were most common due to ease of use and reliability [129]. Isoflurane was infrequently used, likely due to interference with surgical access caused by anesthetic masks. Notably, 71% of studies did not report any postoperative analgesia—an ethical and methodological concern, as unalleviated pain can affect systemic inflammation and healing outcomes [130].

Antibiotic use was inconsistently reported. Of the 30 studies that included antibiotics, most failed to specify the agents or justify their use. Beta-lactams and aminoglycosides were the most frequently reported, in line with standard veterinary practice [131].

Euthanasia timing showed considerable variability, ranging from a few days to over 100 days. The most common time point was 28 days, reflecting an early osseointegration phase [44].

While single-time-point studies focused on endpoint analysis, about one-third employed multiple time points, enabling longitudinal assessment but requiring more animals and resources. Only 11 studies used more than five time points, reflecting the logistical challenges of such designs. An increase in the number of time points resulted in a higher overall sample size, which may compromise compliance with the 3R principles (Replacement, Reduction, and Refinement) [124].

Histological analysis remains the gold standard for evaluating peri-implant tissues. A minority of studies retained implants in situ, despite the importance of analysing bone–implant interfaces. In contrast, implant removal simplified processing and enabled immunohistochemistry by using decalcified samples thus improving antibody penetration and staining quality. Only one study reported performing immunohistochemistry with the implant in place, highlighting the technical limitations of that approach (undecalcified samples).

The use of µCT imaging remains suboptimal [132]. More than half of studies did not utilize this technique. Post-mortem µCT was more frequently adopted and remains the structural imaging gold standard. In vivo µCT was used less often due to technical challenges, including the need for anesthesia and reduced spatial resolution. Nonetheless, in vivo µCT allows longitudinal evaluation within the same animal, reducing inter-animal variability and enabling dynamic observation of bone remodelling [133].

Drop-out rates were generally underreported. Only 22 studies provided mortality data, with a mean mortality rate of 3.35 ± 4.61%, suggesting that surgical implant procedures in rats are generally safe. Implant failure was reported in 29 studies, with an average rate of 10.85 ± 13.47%. The wide range of reported failure rates, from near-zero to over 20%, indicates that certain protocols may carry higher mechanical or biological risks.

This review also highlights important ethical concerns and methodological limitations that emerged across the analysed studies. Notably, 70 studies did not report any analgesia protocol following surgical procedures. While tooth extraction and implant placement may be considered routine in preclinical settings, these interventions are inherently painful, and it is well established that rats are capable of experiencing postoperative pain [134,135]. The omission of analgesia contradicts fundamental animal welfare principles and raises ethical issues regarding study design and animal handling.

Furthermore, only 13 studies reported implementing a dietary adjustment after surgery. Given the high metabolic demands of rodents, postoperative nutritional support is essential to facilitate recovery and maintain animal well-being [136].

Methodological transparency was also frequently lacking. Several studies failed to report essential elements of study design. This lack of transparency undermines the reproducibility of findings and conflicts with the ARRIVE 2.0 guidelines [137], which were developed to improve the standardization and translational relevance of animal research. Particularly, incomplete reporting of complications, such as dropout rates (animal mortality) and implant failure rates, limits the field’s capacity to optimize experimental protocols, refine surgical procedures, and design more ethically robust studies.

Although substantial heterogeneity was observed among the included studies, some methodological features appeared more frequently across the reviewed literature, including the use of Sprague-Dawley rats, maxillary first molar implantation sites, titanium screw-shaped implants, approximately 28-day healing periods, and combined histological and micro-CT evaluation methods. These recurring characteristics do not represent definitive methodological recommendations but may provide a useful reference framework for future preclinical investigations aiming to improve methodological comparability among intraoral rat implant studies.

This scoping review presents some limitations that should be acknowledged. First, the literature search was conducted using only two databases (PubMed and Scopus). Although these represent two of the largest and most comprehensive repositories for biomedical literature, relevant studies indexed elsewhere may have been missed. Additionally, the use of strain-specific keywords within the search strategy may have theoretically limited the retrieval of studies in which less commonly used strains were mentioned only in the title or abstract without corresponding indexing terms.

Second, as a scoping review rather than a systematic one, the aim was to broadly map the current use of intraoral rat models in oral implantology and summarize methodological characteristics across heterogeneous studies. Consequently, no formal quality assessment of individual studies was performed, and variability in study design was not systematically rated.

## 5. Conclusions

This analysis of the literature highlights that the rat oral implant model is feasible, reproducible and informative; that’s why it is increasingly used in preclinical research. The advances in surgical techniques, along with an improved understanding of rat anatomy and physiology, have further supported the establishment of this model as a valuable and widely adopted preclinical platform.

However, this scoping review has also revealed a persistent lack of consensus regarding key trial design features. In particular, adherence to the ARRIVE 2.0 guidelines remains inconsistent across the reviewed studies, thereby limiting methodological transparency and compromising the reproducibility and comparability of findings. These shortcomings limit the ability to conduct robust meta-analyses and impede the advancement of translational implant research.

To improve the scientific value and ethical standards of preclinical studies, future research should prioritize standardized reporting and rigorously designed protocols, thereby enhancing comparability, reproducibility, and ultimately, the clinical relevance of animal data.

## Figures and Tables

**Table 1:** Distribution of the articles according to their Focused Research Questions.

Outcome	Specific Model	Papers
**General disease models**	Osteoporosis	[24,36,37,40,41,50,51,55,68,70,72,82,94,95,96]
	Osteoporosis + antiresorptive medications	[53,58,115]
	Antiresorptive medications	[52,66,81,91,98,99]
	Antiresorptive medications + Stress	[29]
	Diabetes	[18,32,33,49,54,73,84,103,104,109]
	Stress	[97]
	Radiation	[92]
**Local disease models**	Occlusal loading	[19,24,28,31,39,42,45,49,56,65,67,71,100]
	Peri-implantitis	[23,38,62,63,83,110,114]
	Surgery-related bone heating	[35,88]
	Nerve injury	[64,74]
**Different surgical protocols**	Implant surface	[22,23,26,27,37,43,54,55,63,66,78,79,87,90,93,98,100,109,111,114]
	Implant material	[52,85,86,89]
	Implant morphology	[35,101]
	Timing of implant placement	[50]
**Biomaterials**	Collagen sponge	[38,46,76]
	Bone morphogenetic proteins	[80,102]
	Autogenous bone	[30,75,77]
	α-Tricalcium phosphate	[59]
	Hybrid construct	[110]
	Space holder	[69]
**Other Aims**	Studies not fitting predefined categories	[20,21,25,34,44,47,48,57,60,61,105,106,107,108,112,113]

**Table 2:** Distribution of medications administered to establish the experimental models in rats.

Category	Molecule	Papers
**Bone homeostasis** **agents**	Alendronate	[29,58,91,98,99,115]
	Alendronate and Zoledronate	[52,85,86]
	Alendronate and Dexamethasone	[29,98]
	Zoledronate	[66]
	Zoledronate and PTH	[53]
	Parathyroid hormone (PTH)	[70,84,87]
	Raloxifene	[36,51]
	Abaloparatide	[81,82]
**Fluorescent labelling agents**	Calcein	[46,102,104]
	Calcein and Alizarin red S	[55]
	Not specified	[92]
**Medications correlated to Diabetes**	Insulin	[32]
	Metformin	[41]
	Advanced glycation end products (AGEs)	[49]
**Other medications**	Clodronate liposomes	[21,57]
	Simvastatin	[25,95]
	Sclerostin-neutralizing antibodies	[38]
	Botulinum toxin A	[64]
	Liposome WNT3a protein	[30,72]

## Data Availability

No new data were created or analyzed in this study.
